# As above so below: Recent and future advances in plant‐mediated above‐ and belowground interactions

**DOI:** 10.1002/ajb2.1845

**Published:** 2022-04-20

**Authors:** Sergio Rasmann

**Affiliations:** ^1^ Institute of Biology, University of Neuchâtel Rue Emile‐Argand 11, CH‐2000 Neuchâtel Switzerland

**Keywords:** biotic interactions, herbivory, leaf herbivory, plant evolution, plant–soil feedback, root exudates, secondary metabolites, soil community, species interactions

“We were men once, though we've become trees.”

―*Dante Alighieri, Inferno*

I here use Dante Alighieri's quote to set the scene for the scope of this essay: that above‐ and belowground subsystems of any terrestrial ecosystem studied so far are intimately linked and influence each other, mostly through plants as a conduit. Accordingly, the end of the 1990s and the early 2000s saw a surge of research and syntheses on the ecological linkages between above‐ and belowground subsystems (Van der Putten et al., 2001; Wardle et al., 2004b), how they influence each other—mainly through changes in plant traits (Bardgett and Wardle, 2003; van Dam et al., 2003)—and what are the ecological and ecosystem consequences of changes to them (De Deyn and Van der Putten, 2005). Through all of this work, we were able to conclude that aboveground–belowground interactions seem to be widespread—if not omnipresent—in the plant kingdom. However, based on the experimental evidence so far, another major conclusion on this topic is that the magnitude and net effect of these interactions is highly context dependent.

Context dependency on aboveground–belowground interactions can arise from numerous factors. For instance, aboveground herbivory seems to change the activity of soil microbial communities, but this effect can vary over time. Over short periods (days or weeks), foliar herbivory can cause plants to release carbon‐rich molecules into the soil. This observation led to predictions that aboveground herbivory should stimulate the activity of soil microbial communities, which in turn may increase the size of the soil inorganic nitrogen pool available to plants (Hamilton and Frank, 2001). Over longer periods of time, aboveground herbivores may stimulate plant compensatory growth to some extent, which can itself promote further herbivory and increase the return of plant litter, in the form of debris or herbivore feces, to the soil. In turn, such additional litter is generally more labile and more assimilable by the plants than normal plant litter, which can feed back to spur plant productivity (Bardgett and Wardle, 2003). On the contrary, aboveground herbivory can have negative effects on soil communities, if for example foliar herbivores stimulate the production of chemical defences in roots (Bezemer and van Dam, 2005) or over time promote the dominance of unpalatable plant species with poor litter quality (Wardle et al., 2002). Therefore, whether the net effects of the aboveground subsystem on belowground communities via feedback loops on plant productivity are positive or negative clearly depends on context.

Effects of belowground subsystems on aboveground communities can similarly be positive or negative. Root herbivory can cause a reduction in plant growth or yield (Blossey and Hunt‐Joshi, 2003), while beneficial soil microbes and fungi can directly stimulate plant growth and nutrient acquisition (Dellagi et al., 2020). Accordingly, reviews on below‐to‐aboveground processes generally show that the effects of soil microbial and animal communities on aboveground plant community productivity, composition, and diversity can range from positive to negative depending on context (Johnson et al., 2012). Therefore, while the net effects of aboveground–belowground subsystems have received some scientific attention, our ability to generalize requires further investigations, but also a better understanding of the mechanisms behind these effects (Wardle et al., 2004a). For instance, within the mechanistic scope, several studies on plant‐mediated aboveground‐belowground interactions have applied hypotheses borrowed from the plant defence theory framework (Bezemer and van Dam, 2005), which allowed formulating predictions on the proportional contribution of plant‐specialized compounds versus primary metabolites to plant–herbivore interactions above‐ or belowground.

**Figure 1:**
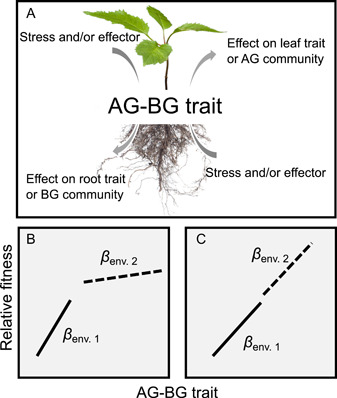
Hypothetical strengths of natural selection on the aboveground–belowground (AG‐BG) trait across two different environments. Panel A depicts an AG‐BG trait that can be measured on individual plants. AG‐BG could be measured by imposing a standardized effector/stressor on leaves/roots and measuring a response on roots/leaves. Panels B and C show selection gradients (*β*) acting on the strength of aboveground–belowground interactions as the line of best fit between relative fitness in a population and the AG‐BG trait. Consider two populations of the same species: one in environment 1 (solid line) and one in environment 2 (dashed line). In scenario B, the evolutionary outcome of this interaction (assuming all else to be equal) is driven by the difference in the level of aboveground or belowground interaction because there is a nonlinear relationship between relative fitness and the strength of selection on AG‐BG trait, such that *β*_env1_ < *β*_env2_. Thus, all else being equal, the rate of evolution for the AG‐BG trait will be faster in environment 1. In scenario C, plants on average have lower fitness in environment 1, and lover levels of the AG‐BG trait than in environment 2. In this scenario, the strength of selection acting on the two populations is identical (*β*_env1_ = *β*_env2_). Thus, all else being equal, the direction, rate, and long‐term consequences of evolution by natural selection will be the same in the two environments.

To summarize the above, studies of ecological linkages between above‐ and belowground communities appear to have taken two parallel routes. The first route has focused on the quality and quantity of resources (mainly carbon and nutrients) that the plant produces both above‐ and belowground and how these resources enter the soil, are degraded, and feed back to plant growth. The second has focused on the mechanisms of such interactions and, more specifically, how plant physiological changes, such as plant defence strategies or the production of particular primary and secondary metabolites, mediate above‐ and belowground interactions between herbivores and higher trophic level organisms (Soler et al., 2005). Here, I argue that one key route is largely missing from this roadmap: evolution. We now know that aboveground–belowground interactions are species‐specific and that they vary depending on local ecological conditions (i.e., they are plastic). At the same time, there is mounting evidence that aboveground–belowground interactions are genetically based and that there is natural genetic variation in how plants respond to above‐ or belowground herbivore and pathogen attack in shoots versus roots and vice versa (Singh et al., 2014). For instance, van Dam and Vrieling (1994) by applying standardized artificial damage to half‐sibs of *Cynoglossum officinale*, they showed that about half of the genetic families increased pyrrolizidine alkaloids in their shoots, and the others in their roots, thus showing that there is genetic variation for inducibility across organs. In another example, half‐sib families of *Cardamine hirsuta* induced with the phytohormone jasmonic acid in the roots differed in how they induced glucosinolates in their leaves, and ultimately how many seeds they could produce after *Pieris brassicae* caterpillar leaf feeding (Bakhtiari and Rasmann, 2020). Together, these findings imply that there is a raw substrate for natural selection to act upon in either strengthening or decreasing plant‐mediated aboveground–belowground interactions. In other words, if we consider the strength of plant‐mediated aboveground–belowground interactions to be a trait that can be measured on any given plant genotype or individual (hereafter AG‐BG), by correlating AG‐BG with plant fitness‐related traits (e.g., fruit production, seed production, growth rate) we can ultimately measure the strength of selection on it (Figure 1). The next logical question in this regard is: Which environments do we expect to favor the evolution of a strong linkage between the two subsystems? This question is clearly both a mechanistic and a population‐ or community level question that needs ecosystem‐level information. However, to my knowledge, only a few attempts have been made to investigate the consequences of above‐ and belowground stressors, such as herbivory, on plant fitness in natural settings and across variable environments (Barber et al., 2011). Embedding environmental variation into aboveground–belowground studies, for instance through ecological gradients, can help to understand the boundaries of such interactions, such as those driven by resource limitation. For instance, biotic and abiotic factors can differ dramatically and in opposition at the low and high ends of elevational gradients; at low elevation, biotic stress should be maximal, while at high elevation abiotic stress should be maximal. Therefore AG‐BG constitutive state versus inducibility should vary along elevation in a predictable manner. For instance, if either or both leaf and root herbivory is high and constant, plants should invest in high constitute defences in the respective organs, but low levels of AG‐BG. On the contrary, at intermediate and less predictable levels of herbivory, for instance, at mid elevation, plants should invest in high AG‐BG inducibility (Kergunteuil et al., 2018). Approaching the question of AG‐BG variability could therefore benefit from taking any ecological gradient approach, such as studying these interactions within natural gradients in resources and abiotic or biotic stressors.

Moreover, to approach the conundrum of the evolutionary route to aboveground–belowground interactions is to detail a standardized method to measure AG‐BG—as has been done for other plant functional traits (Pérez‐Harguindeguy et al., 2013) (Figure 1A). For instance, one could imagine imposing a fixed rate of leaf herbivory on the same plant species placed in different environments, and then measuring the concentration of sugars, secondary metabolites, and/or exudates in the roots. Vice versa, one could add phytohormone solutions or other effectors to the roots and then measure phytochemical changes in leaves and variation in herbivore performance. Finally, a fitness‐related trait (e.g., fruit production, flower number, biomass) should be measured alongside AG‐BG. These types of experiments are more easily performed in controlled common garden settings for detecting the genetic basis of variation, but with appropriate controls, they could also be implemented in realistic field conditions for detecting genotype‐by‐environment interaction, and the relative contribution of different ecological factors driving AG‐BG trait selection.

In sum, as Dante already hinted at, plant‐mediated aboveground–belowground interactions can have widespread effects on community structure, population dynamics, and ecosystem functioning by connecting two otherwise separated subsystems. Research so far has highlighted that there is a high degree of taxonomic and ecological context dependency in such interactions and a relatively high amount of natural genetic variation among plant species. How such variation is maintained across sites and whether AG‐BGs are subject to different selection pressures depending on local ecological conditions (Figure 1B, C) should be studied further. I argue that the best way to achieve this goal is by developing a standardized way of measuring a novel AG‐BG trait to the growing list of plant functional traits, and also develop testable hypotheses—as discussed above—that address for instance how plant strategies change as a function of how different rates of above‐ and belowground herbivores, and depending on the feeding mode, the abundance and frequency of attacks and the specialization of the herbivore (Adler and Karban, 1994).
